# Studies on the role of non-coding RNAs in controlling the activity of T cells in asthma

**DOI:** 10.1016/j.ncrna.2023.02.004

**Published:** 2023-02-15

**Authors:** Albert Sufianov, Marina Bessonova, Sema Begliarzade, Valentin Kudriashov, Andrei Danilov, Tatiana Ilyasova, Wang Yaolou, Radmila Nafikova, Ozal Beylerli

**Affiliations:** a Educational and Scientific Institute of Neurosurgery, Рeoples’ Friendship University of Russia (RUDN University), Moscow, Russia; b Department of Neurosurgery, Sechenov First Moscow State Medical University (Sechenov University), Moscow, Russia; c Tyumen Cardiology Research Center, Tomsk National Research Medical Center, Russian Academy of Science, Tomsk, Russia; d Republican Clinical Perinatal Center, Ufa, Republic of Bashkortostan, 450106, Russia; e Gastric Cancer Center, West China Hospital of Sichuan University, China; f Department of Clinical Pharmacology, Smolensk State Medical University, Smolensk, Russia; g Department of Internal Diseases, Bashkir State Medical University, Ufa, Republic of Bashkortostan, 450008, Russia; h Harbin Medical University, 157 Baojian Rd, Nangang, Harbin, Heilongjiang, 150088, China; i Department of Pulmonary and Critical care, Northwestern University, Chicago, United States

**Keywords:** Non-coding RNAs, Circular RNAs, Long non-coding RNAs, microRNAs, Asthma, T cells

## Abstract

Bronchial asthma, commonly known as asthma, is a chronic inflammatory disease characterized by airway inflammation, increased responsiveness and changes in airway structure. T cells, particularly T helper cells, play a crucial role in the disease. Non-coding RNAs, which are RNAs that do not code for proteins, mainly include microRNAs, long non-coding RNAs, and circular RNAs, play a role in regulating various biological processes. Studies have shown that non-coding RNAs have an important role in the activation and transformation of T cells and other biological processes in asthma. The specific mechanisms and clinical applications are worth further examination. This article reviews the recent research on the role of microRNAs, long non-coding RNAs and circular RNAs in T cells in asthma.

## 1 Introduction

Asthma is one of the common chronic diseases in my country and even in the world. According to statistics from the Global Initiative for Asthma (GINA), the number of people with asthma worldwide has exceeded 300 million, and the incidence of the disease is increasing year by year. It poses a huge threat to the safety of human life and imposes a heavy psychological and economic burden on families and society (https://ginasthma.org/, 2020). With the development and application of a variety of therapeutic drugs and the continuous improvement of treatment methods, the symptoms of most asthma patients can be clinically relieved or controlled, but 5%–10% of patients still have recurrent asthma symptoms, often accompanied by airway inflammation Infiltration and remodeling can lead to death from respiratory distress [1]. Current studies have shown that the pathogenesis of asthma is closely related to the imbalance of T cell subsets and their cytokines, such as the imbalance among each subset of helper T cells (Th1, Th2, Th9, Th17, Treg, etc.) and the cytokines secreted by them. disorder [[2], [3], [4], [5]]. Non-coding RNAs (ncRNAs) is a class of protein non-coding molecules that widely exist in eukaryotes, participate in a variety of biological regulation processes, and have been widely studied in human diseases [[6], [7], [8], [9], [10], [11]]. It mainly includes microRNAs (miRNAs), long non-coding RNAs (lncRNAs), circular RNAs (circRNAs) and so on. Recent research results show that ncRNAs may play an important role in the inflammatory process mediated by asthmatic T cells, in which miRNAs and lncRNAs can regulate the activation, proliferation, apoptosis, transformation and secretion of cytokines of T cells, etc [2,[12], [13], [14], [15]]. However, little is known about the role of circRNAs in the pathogenesis of asthma. This article reviews the latest research progress on the regulatory function and molecular mechanism of ncRNAs (miRNAs, lncRNAs, circRNAs) in asthma T cell-mediated inflammation, providing new ideas for better understanding the pathogenesis of asthma and improving the diagnosis level. It also provides a theoretical basis for exploiting the regulatory potential of ncRNAs to develop therapeutic strategies.

## 2 Introduction to T cell classification and function

T cells are complex and diverse, with subpopulations that can have different developmental stages or functions present in the body at the same time. At present, there are various ways to classify T cells. They can be divided into two subgroups, CD4^+^ and CD8^+^, based on their cell surface differentiation antigen (CD); or they can be classified based on their functions as helper T cells (Th cells), suppressive T cells (Ts cells), delayed type hypersensitivity T cells (TDTH cells) and cytotoxic T cells (CTL or Tc cells). Th cells belong to CD4^+^ T cells and are further divided into subgroups such as Th1, Th2, Treg, Th17, Th9, Tfh and others, based on their surface markers, transcription factors and secreted cytokines. Th1 produces IL-2, TNF-α, IFN-γ and so on; Th2 produces IL-4, IL-5, IL-9, IL-10, IL-13, and so on; Th17 produces IL-6, IL-17A, IL-22 and TNF-α, and so on; Treg produces IL-10, TGF-β and other cytokines [[16], [17], [18]]. Current studies have shown that the development and transformation of Th cell subsets and the expression of their specific genes is controlled by a complex network of transcription factors, epigenetic changes, and post-transcriptional regulators. The development of polarized Th cells is the core of the pathogenesis of allergic inflammation, because allergic inflammation is mainly a Th2 response. Th2 plays a major role in allergic inflammation of asthma and other subgroups such as Th1, Treg, Th17, Th9, and Tfh also play an important role in it [[19], [20], [21], [22], [23], [24], [25], [26], [27], [28]].

## 3 Introduction to NCRNAS classification and function

NcRNAs plays a significant role in regulating important processes such as cell differentiation and development, and is closely related to the onset and progression of diseases [29]. They can be divided into two categories based on their functions: housekeeping ncRNAs and regulatory ncRNAs. Housekeeping ncRNA includes molecules such as ribosomal RNA, small nucleolar RNA, small nuclear RNA, transfer RNA, guide RNA and telomerase RNA. Regulatory ncRNAs includes short ncRNAs (less than 200 nucleotides, such as microRNAs) and long ncRNAs (more than 200 nucleotides, such as long non-coding RNAs, and circular RNAs) [30].

MiRNAs is a single-stranded ncRNAs composed of about 19–25 nucleotides, which exists in animals and plants and has a high degree of conservation in the process of biological evolution. let-7 is the most abundant class of miRNAs, and it is also the miRNAs discovered before the miRNAs nomenclature was established. The let-7 family has 13 members: let-7a-1, let-7a-2, let-7a-3, let-7b, let-7e, let-7d, 1et-7e, let-7f-1, let -7f-2, let-7g, let-7i, miR-202 and miR-98, each derived from 9 different chromosomes, but the "seed sequence" (nucleotide sequence TGAGGTA at the 5′ end) of all members of the family Highly conservative. miRNA mainly guides the RNA-induced silencing complex (RISC) to down-regulate the expression of target genes through one of the two silencing mechanisms of mRNA cleavage or translational repression [31].

**Fig. 1:**
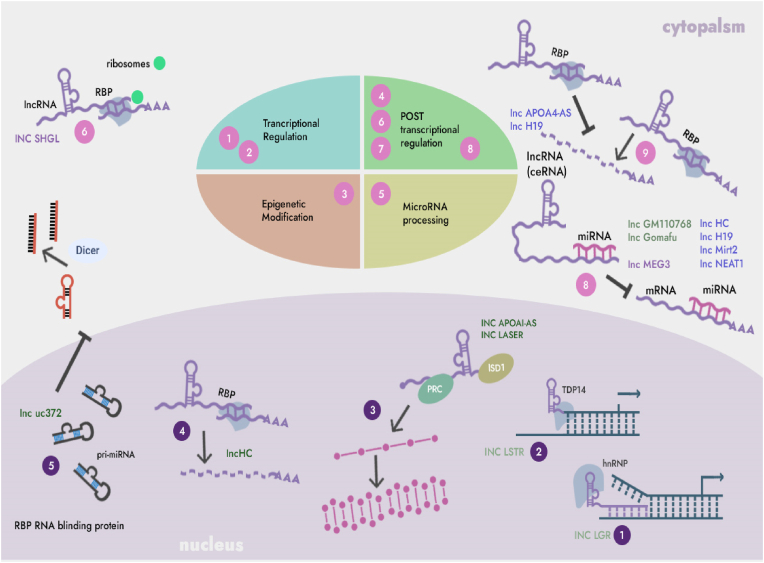
Schematic diagram of the mechanism of action of lncRNAs. Nucleus: (1) Binds to nucleoproteins and affects chromatin state. (2) Binding to nucleic acid binding proteins to regulate gene expression. (3) Affect miRNA maturation and release. (4) Combine with RNA binding protein to change the state of RNA after transcription. Cytoplasm: (1) Binding to RNA binding proteins, affecting mRNA translation efficiency and stability. (2) with iRNA binding prevents it from interacting with target gene mRNA.

LncRNAs is an ncRNAs with a length of more than 200 nucleotides, which is less conserved in the process of biological evolution, but has high cell and tissue specificity. LncRNA plays an important role in various life processes such as immune-inflammatory response by acting as a signal, decoy, guide or scaffold molecule to regulate gene expression at the transcriptional level (Fig. 1) [32].

CircRNAs is a special kind of ncRNAs molecule that forms a circular structure with covalent bonds, without a 5′ end cap and a 3′ end poly(A) tail. It is not affected by RNA exonucleases and has a high degree of abundance and evolutionary conservation. CircRNAs acts as a "sponge" to adsorb miRNA, interact with proteins, regulate gene splicing or transcription, protein or polypeptide translation, and epigenetics to participate in various biological processes [33].

The above three ncRNAs can interact to exert some of their functions. LncRNAs and circRNAs can adsorb miRNA through miRNA response elements (MREs), forming a competitive endogenous RNA (competing endogenous RNA, ceRNA) network to exert their biological effects [34].

## 4 MIRNAS is involved in the regulation of asthma T cell function

Accumulating evidence indicates that miRNAs are important regulators of immune system development and function, and play an important role in regulating Th cell activation, differentiation, and cytokine secretion [2,[35], [36], [37], [38], [39], [40], [41], [42], [43], [44], [45], [46]]. At present, there are more and more researches on the role of miRNA in the inflammatory process of asthma by regulating the function of T cells. secretion, etc. are involved in the inflammatory process of asthma (Table 1) [[47], [48], [49], [50], [51], [52]].

**Table 1:** MiRNAs in regulation of T cell function in asthma.

miRNA	Cell type	Targets	Function	Reference
let-7	T cells	IL-13	Downregulates IL-13	[48,53]
let-7f	Th17	IL-23R, IL-17A	Inhibits expression of IL-23R and IL-17A	[54]
miR-106a	RAW264.7	IL-10	Downregulates IL-10	[47]
miR-323–3p	Th17	SMAD, SMAD3, IL-22	Modulates TGF-β-dependent signaling pathway and	[55]
miR-155	CD4^+^ T	c-Maf, IL-4/5/13	Inhibits the Th2 differentiation *in vitro*; promotes DC-induced activation of Th2 cells *in vivo*	[50,56,57]
	T cells	CTLA-4	Enhances proliferative response of T cells	[58]
miR-21	Dendritic cells	IL-12p35 3′UTR	Modulates Th1/Th2 polarization	[59]
miR-17–92	T cells	PTEN, SOCS1, A20	Increases Th1, Th2 and Th17 responses	[60,61]
miR-146a	T cells	STAT1	Enhances the inhibition of Th1 response mediated by Treg cells	[62]
miR-126	Airway wall cells	OBF.1 or BOB.1	Increases Th2 response	[49]
miR-145	CD4^+^ T	RUNX3	Modulates the Th1/Th2 balance	[63]
miR-29c	Macrophage	B7–H3	Inhibits the Th2/Th17 differentiation	[64]
miR-192	CD4^+^ T	CXCR5	Inhibits the Tfh differentiation	[65]

### 4.1 The role of a single miRNA or miRNAs of the same gene cluster in the function of asthmatic T cells

#### 4.1.1 Mainly involved in regulating the secretion of cytokines

In an allergen-induced mouse asthma model, high-throughput sequencing of lung tissue revealed that a large number of miRNAs were differentially expressed, among which the let-7 family was highly expressed in the lung [66,67]. Studies by Polikepahad et al. showed that let-7a is more highly expressed in Th1 cells compared to Th2 cells, and by using let-7a/b/c/d antagonists to silence the above miRNAs; Inflammatory cells were reduced in alveolar lavage fluid of asthma model mice, and the levels of IL-4, IL-5 and IL-13 were all reduced, however, this was contrary to the results of in vitro experiments [53]. Another study showed that the expression of let-7 family members in the lung tissue of mice stimulated with ovalbumin (OVA) was down-regulated. After nasal administration of let-7 mimics, the airway inflammatory cell infiltration, mucus secretion, airway fibrosis and airway hyperresponsiveness in mice were all alleviated. In vitro cell experiments confirmed that let-7 inhibited polyacrylic acid Methyl ester/phytohemagglutinin (PMA/PHA)-induced secretion of IL-13 in T cells [48]. The route and frequency of administration of the two studies, the difference in the use of drugs, and the off-target of miRNA *in vivo* may be the main reasons for the inconsistent experimental results.

The studies described above indicate that let-7 family members appear to target the expression of IL-13, thereby modulating Th2-type responses. The study by Newcomb et al. showed that estrogen and progesterone decreased the expression of let-7f in patients with severe asthma, and let-7f could inhibit the expression of IL-23R and the production of IL-17A in Th17 cells [54]. In summary, the let-7 family may participate in the inflammatory mechanism of asthma by regulating the secretion of T cell-related cytokines.

In addition, miR-323–3p inhibits Th17 cells from secreting IL-22 [55]; miR-106a is up-regulated in the lungs of OVA-treated mice, and inhibiting miR-106a can up-regulate the expression of IL-10 [47,68]. It is worth noting that the administration of miR-106a antagonists after intranasal allergen challenge can still significantly reduce asthma phenotypes such as inflammatory cell infiltration and Th2 cytokine levels, so it may be highly relevant to the treatment of clinical diseases.

#### 4.1.2 Mainly involved in regulating the activation and differentiation of T cells

MiR-155, a crucial immunoregulatory factor, can promote the proliferation of T cells and inhibit cytotoxic T lymphocyte-associated protein 4 (CTLA-4), and regulate the activation and differentiation of Th2 cells, thereby playing a role in the development of asthma [50,58,69,70]. Studies have shown that the loss of miR-155 can relieve the repression of the c-Maf gene and cause Th cells to exhibit Th2 bias in vitro [69]. However, other studies have found that miR-155 is upregulated in OVA-induced mouse lung tissue and spleen CD4^+^ T cells [50,56,71]. These contradictory findings may be due to the different sources of cells used in the experiments. Further research is needed to fully understand the unique role of miR-155 in the development of Th2 cells and its potential as a therapeutic target in the treatment of allergic airway inflammation.

MiR-21 was significantly elevated in the lungs of mice exposed to both HDM and OVA [], and miR-21 is involved in the inflammatory mechanism of asthma by inhibiting Th1 differentiation and enhancing Th2 polarization [49,51,59,72]. Since the 3′UTR of IL-12p35 contains a highly evolved and conserved miR-21 target sequence, miR-21 may regulate the conversion of Th1 to Th2 phenotype by degrading the transcript of IL-12p35 [51]. Inhibition of miR-21 led to downregulation of CD4+/CD8− T cell ratio and Th2 cytokine levels in the spleen of asthmatic model mice, and this process directly targeted IL-12p35 through miR-21 [59,73]. However, in another HDM-induced asthma model, intranasal administration of miR-21 antagonists after intranasal sensitization before allergen challenge had no significant effect on Th2 cytokine production, which may be due to the balance of Th1 and Th2 at the time of administration. established, indicating that miR-21 played the most important role in the early sensitization stage, and miR-21 may be an effective early therapeutic target for asthma [74].

The miR-17–92 cluster includes six mature miRNAs including miR-17, miR-18, miR-19a, miR-19b, miR-20, and miR-92 can be involved in lymphoproliferative and autoimmune diseases by regulating T cell function [61,75]. MiR-17–92 was significantly upregulated in peripheral blood CD4^+^ T cells of asthmatic patients, among which miR-19a enhanced Th2 type response by targeting PTEN, SOCS1 and deubiquitinase A20 [60]. LncRNA-MEG3, as a ceRNA, regulates Treg/Th17 balance in asthmatic patients by targeting miR-17/RORγt [12]. In a mouse model of allergic airway inflammation, the miR-17–92 cluster affected the homeostasis of ILC2s and the expression of IL-5/13 by repressing SOCS1 and A20 genes [76]. In summary, miR-17–92 plays an important role in the inflammatory mechanism mediated by T cells in asthma.

Lu et al. found that miR-146a deficiency selectively attenuates Treg cell suppression of Th1 responses, but not Th2 and Th17 responses, in part by targeting Signal Transducer and Activator of Transcription 1 (STAT1) [62]. MiR-146a and miR-146b were upregulated in splenic CD4^+^ T cells of an OVA-induced mouse asthma model, while miR-146a was downregulated after dexamethasone treatment [71]. A study of patients with severe asthma showed that both miR-146a and miR-146b were decreased in CD4^+^ T and CD8^+^ T cells [15]. Downregulation of miR-146a may partly mediate the severe asthmatic phenotype, as T cells lacking miR-146a have been found to be hyperactive in both acute and chronic inflammatory states [77]. Although the specific role of miR-146b in the regulation of adaptive immune responses has not been investigated, miR-146a and miR-146b share the same seed sequence, which is critical for miRNA-mediated target gene expression, so further studies are needed to determine whether miR-146b plays a role in asthma.

In addition, some studies have also shown that miRNA is involved in the activation and differentiation of T cells in the pathogenesis of asthma. For example, the expression of miR-126 is up-regulated in asthma models, and the expression of POU domain type 2 binding factor 1 (OBF.1 or BOB.1) is up-regulated after miR-126 is blocked, which can inhibit the expression of transcription factor GATA3 by activating transcription factor PU1, inhibiting Th2 type response [49]. The expression of miR-145 was upregulated in the lungs of HDM-treated mice, and inhibition of miR-145 could lead to a decrease in the levels of IL-5 and IL-13 [74]. MiR-145 can also affect Th1/Th2 levels in asthmatic patients by regulating the Runx3 gene. balance [63]. MiR-29c/B7–H3 (costimulatory molecule) plays an important role in childhood asthma by regulating the differentiation of Th2/Th17 cells [64]. MiR-192 inhibits Tfh differentiation in childhood asthma by targeting chemokine receptor 5 [65].

### 4.2 The role of multiple miRNAs derived from different gene clusters in the function of asthmatic T cells through synergy or mutual antagonism

Three recent studies have further strengthened our understanding of the molecular mechanism of miRNA involvement in asthma: five Th2-related miRNAs (miR-27b, miR-206, miR-106b, miR-203, miR-23b) antagonize each other leading to significantly lower Th2 response [78]. MiR-371, miR-138, miR-544, miR-145 and miR-214 regulate the Th1/Th2 balance in asthma through the combined regulation of the Runx3 gene [79]. Two miRNAs, miR-24 and miR-27, are co-expressed in two gene clusters, and they can both independently inhibit IL-4 production and synergistically inhibit Th2 responses, indicating that multiple miRNAs can cooperate or interact with each other [66]. Antagonism to participate in the regulation of asthmatic T cell function.

### 4.3 Other

The expressions of miR-181a and miR-150 in spleen CD4^+^ T cells of OVA-induced mouse asthma model were upregulated [71]; miR-11 in sorted CD4^+^ T and Th2 cells in acute asthma mouse model was significantly reduced, and CD4^+^ T cells miR-295–3p and miR-294–3p are up-regulated, while miR-375–3p and miR-2137 are down-regulated [80]; miR-93, miR-181a, miR-26a and miR-874 were down-regulated in Th17 cells of children with asthma [55]. The aforementioned miRNAs are differentially expressed in asthmatic T cells, but their molecular mechanisms have not been investigated.

## 5 LNCRNAS is involved in the regulation of asthma T cell function

Current studies have shown that some lncRNAs participate in immune regulation by regulating the development of T cells [[81], [82], [83]]. Th1-specific lncRNAs include IFNG-AS1 and linc-MAF-4. IFNG-AS1 is involved in Th1 differentiation [82]. Knocking out linc-MAF-4 in CD4^+^ T cells activated under non-polarizing conditions reduced the expression of Th1 family-specific mRNA [83]. Th2-specific lncRNAs include linc-Ccr2-5′AS, TH2LCRR and GATA3-AS1. Loss of Linc-Ccr2-5′AS results in loss of Ccr1, Ccr2, Ccr3, and Ccr5 [84]. GATA3-AS1 is highly expressed in CD4^+^ T cells [84]. Loss of TH2LCRR can downregulate human Th2-type cytokines [85]. LncRNA DQ786243 regulates the differentiation of Treg cells by affecting the expression of Treg-related cyclic adenosine monophosphate response element binding protein and Foxp3 [10]. The above studies all show that lncRNA plays an important role in the immune regulation involving T cells. However, there are still relatively few studies on it in asthma, as summarized in Table 2.

**Table 2:** LncRNAs in regulation of T cell function in asthma.

lncRNA	Cell type	Methods	Targets	Reference
MEG3	CD4^+^ T	Sequencing and qRT-PCR	miR-17/RORγt	[12]
lnc000127	PBMCs, CD4^+^ T	Sequencing and qRT-PCR	CCR8, CRLF2, CD40L	[86]
RP11–401.2	PBMCs	Sequencing and qRT-PCR	–	[88]
MALAT1	CD4^+^ T	qRT-PCR	miR-155/CTLA-4	[89]
fantom3-9230106C11	CD4^+^ T	Sequencing and qRT-PCR	–	[90]

Differentially expressed lncRNAs profiles were found in both CD4^+^ T and CD8^+^ T cells of asthmatic patients and CD4^+^ T cells in the spleen of asthmatic mice [12,15,[86], [87], [88],^90^,^91^]. It was further verified that LNC_000127 was highly expressed in eosinophilic asthma, and knockdown of LNC_000127 in PMA/CD28-activated T cells decreased CCR8, CRLF2 and CD40L (Th2 inflammatory receptors).

It was further verified that LNC_000127 was highly expressed in eosinophilic asthma, and the knockout of LNC_000127 in PMA/CD28-activated T cells decreased the expressions of CCR8, CRLF2 and CD40L (Th2 inflammatory receptors). It is suggested that LNC_000127 is a positive regulator of Th2 inflammation induced by PMA/CD28 [86]; Zhu et al. confirmed that the expression of RP11–401.2 was up-regulated in whole blood of patients with eosinophilic asthma [88]. LncRNA fantom3_9230106C11 was found to be significantly down-regulated in CD4^+^ T cells and Th2 cells in an acute asthma model [90]. Another asthma model study found that MM9LINCRNAEXON12105+ and AK089315 were up-regulated in asthma models, and down-regulated when treated with iPSC-MSCs, suggesting that lncRNAs play a role in iPSC-MSCs-mediated alleviation of asthma Th2 inflammation [91]. The expressions of lncRNA-MEG3 and 18 other lncRNAs were significantly changed in CD8^+^ T cells of patients with severe asthma, and 5 lncRNAs were differentially expressed in CD4^+^ T cells [15]. Recent studies have also shown that lncRNA-MEG3 can "sponge" adsorb miR-17 in CD4^+^ T cells of asthmatic patients, thereby regulating the expression of RORγt and ultimately affecting the balance of Treg/Th17, MALAT1 regulates the expression of CTLA-4 by adsorbing miR-155 through the "sponge" to participate in the regulation of Th1/Th2 balance in CD4^+^ T cells, suggesting that lncRNA/miRNA may have potential application value in the clinical treatment and diagnosis of asthma [12,89]. Therefore, this research group analyzed the lncRNA profile in peripheral blood CD4^+^ T cells of asthmatic patients, and found that the expression levels of three lncRNAs, namely ENST-0000044468, ENST00000566098 and ENST00000583179, were up-regulated, while ENST00000579468 was down-regulated. And there is a good correlation between these lncRNAs and clinical data [87]. This study enriches the content of lncRNA in the regulation of asthma T cell function.

## 6 CIRCRNAS is involved in the regulation of asthma T cell function

As a special class of ncRNAs molecules, circRNAs has become a new research hotspot in recent years. Studies have shown that circRNAs may play an important role in T cell development, and can function as ceRNA of miRNA. For example, the LPS-induced circRNA-mcircRasGEF1B participates in the immune regulation of the body by regulating the stability of intercellular cell adhesion molecule-1 (ICAM-1) mRNA [92]. Analysis of circRNAs in each subpopulation of cells revealed differences in their expression profiles, and further studies showed that the downregulation of hsa_circ_0012919 led to DNA methylation of CD11a and CD70 in CD4^+^ T cells [[93], [94], [95], [96]]. CD28-mediated circRNA100783 is upregulated during CD8^+^ T cell senescence [94]. Hsa_circ_0045272 may negatively regulate T cell apoptosis and IL-2 secretion through the "sponge" adsorption of hsa-miR-6127 [95]. CircIKZF1, circTNIK, circTXK and circFBXW7 are T cells-specifically expressed circRNAs [96]. In the context of this research, we speculate that circRNAs may also have a potential regulatory function in the process of asthma T cell-related inflammation, but there is no related research so far. Therefore, this research group analyzed the circRNAs profile in CD4^+^ T cells of asthmatic patients, and the results showed that compared with healthy volunteers, there are a large number of differentially expressed circRNAs in CD4^+^ T cells of asthmatic patients, and hsa_circ_0005519 can adsorb hsa through the "sponge" -let-7a-5p and affect the secretion of IL-13/IL-6, and ultimately participate in the inflammatory process mediated by asthmatic T cells [97]. This study provides a new idea for the pathogenesis of asthma.

## 7 Conclusion

NcRNAs has a wide range of functions, and each ncRNA has its own special function [[98], [99], [100], [101], [102]]. This article mainly summarizes the role of miRNA, lncRNA and circ-RNA in the regulation of T cell function in asthma. The role of miRNA in regulating the inflammatory mechanism mediated by asthmatic T cells is mainly reflected in several aspects: regulating the differentiation and development of T cells; regulating the activation state of T cells; promoting or inhibiting the transcription of inflammatory genes. The pathway of regulation can be that a single miRNA regulates one or more mRNAs, or that multiple miRNAs of one or more gene clusters act cooperatively on one or more mRNAs to exert biological effects, or interact with other ncRNAs to form a complex regulatory network. However, the association of multiple miRNAs and the mechanism of ceRNAs composed of other ncRNAs (lncRNAs and circRNAs) are less studied. LncRNAs participates in the regulation of asthma T cell-mediated inflammatory mechanism, similar to miRNA, acting on different T cell subsets (Th1, Th2, Treg) to affect their activation, transformation and cytokine secretion. Most of the existing studies only analyzed their expression profiles and identified differentially expressed lncRNAs molecules, but did not conduct in-depth studies on their precise molecular mechanisms. CircRNAs may play an important role in the development of T cells, and may also play a potential regulatory function in the inflammation process mediated by asthma T cells. It can be used as ceRNAs of miRNA to exert its biological effects, but the specific mechanism is still unclear. At present, there is no other related research except that our research group analyzed the circRNAs expression profile in peripheral blood CD4^+^ T cells of asthmatic patients and discovered and reported the molecular mechanism of hsa_circ_0005519 involved in T cell-mediated inflammatory process [97].

Despite the high incidence of asthma, so far, there are few studies on how miRNAs regulate the function of asthmatic T cells. Given the types of miRNAs and their complex regulatory networks, the current research is just the tip of the iceberg; there are even fewer studies on lncRNAs and circRNAs in the process of asthma. Combined with the competition mechanism between ncRNAs, it is speculated that these two types of ncRNAs may have potential significance for the research and application of asthma.

In summary, three ncRNAs, miRNAs, lncRNAs and circRNAs, play an important role in the regulation of T cell function in asthma. However, the molecular mechanism of ncRNAs or its ceRNAs in the regulation of asthma T cell function remains to be further studied. At present, most studies use RNA sequencing technology to discover a large number of differentially expressed ncRNAs, but their molecular mechanisms are poorly understood. Therefore, how to find out the functional ncRNA and clarify its precise molecular mechanism is the difficulty and challenge of ncRNA research in this field. It is believed that with the deepening of research, more and more ncRNAs involved in the pathogenesis of asthma will be discovered, and their role in the inflammatory process mediated by asthma T cells will be revealed, providing new ideas and theoretical basis for the pathogenesis of asthma. In order to develop new markers and targets for the diagnosis, typing and treatment of asthma.

## Funding

This work was supported by the Bashkir State Medical University Strategic Academic Leadership Program (PRIORITY-2030).

## Author contributions

Albert Sufianov, Marina Bessonova and Sema Begliarzade conceptualized and designed the study. All authors have participated in the acquisition, analysis and interpretation of the data. Radmila Nafikova, Andrei Danilov and Valentin Kudriashov has drafted the manuscript. Tatiana Ilyasova and Wang Yaolou contributed to the critical revisions of the manuscript. Ozal Beylerli supervised the research. All authors agreed on the journal to which the article would be submitted, gave the final approval for the version to be published, and agreed to be accountable for all aspects of the work.

## Declaration of competing interest

The authors declare they have no conflict of interest.
